# Management of Bladder Pain Syndrome (BPS): A Practical Guide

**DOI:** 10.1155/2022/7149467

**Published:** 2022-01-10

**Authors:** Patrick Juliebø-Jones, Karin M. Hjelle, Jannike Mohn, Gigja Gudbrandsdottir, Ingunn Roth, Adeel Asghar Chaudhry, Anne Kvåle Bergesen, Christian Beisland

**Affiliations:** ^1^Department of Urology, Haukeland University Hospital, Bergen, Norway; ^2^Department of Clinical Medicine, University of Bergen, Bergen, Norway

## Abstract

Bladder pain syndrome (BPS) is a prevalent and pervasive disease. The physical and psychological sequelae can be very burdensome for the patient, and the condition represents a real challenge for the clinician as well. With no simple pathognomonic test, finding harmony in navigating patient care can be demanding. Diagnosis and management rely upon a multidisciplinary and holistic approach. Treatment options include conservative measures and pharmacotherapies as well as bladder instillation therapies. Ultimately, surgery may be offered but only in cases of refractory disease. This article offers a pragmatic guide for clinicians managing this challenging disease.

## 1. Manuscript

Bladder pain syndrome (BPS) is a disease of ubiquity, and epidemiological studies estimate it to affect 1.8–51/10,000 individuals worldwide [1]. However, there is evidence to suggest that the prevalence is underreported and that less than 10% of disease sufferers receive a formal diagnosis [2]. Prior to recent changes in standardised terminology, BPS was formerly referred to as interstitial cystitis (IC) [3]. This shift in nomenclature reflects its multifactorial aetiology and the symptom-based approach, which now occupies the diagnostic workup. The burden of the BPS is substantial, regarding both physical and psychological sequelae. It has been highlighted by the World Health Organization (WHO) as a major public health issue [4, 5]. Difficulties in diagnosing the condition are mirrored by the challenges faced in treating it. Despite the availability of an abundance of novel treatments, most of these have a limited evidence basis to support their use [6]. Such is the diversity in clinical phenotypes associated with BPS, and there is a huge range in treatment strategies offered worldwide. While numerous international guidelines do exist, recommendations can be divergent [7, 8]. It can therefore be quite difficult for the clinician to find harmony in navigating patient care. Our aim was to provide an overview of a practical framework for the contemporary management of BPS.

## 2. Methods

The diagnostic and treatment pathways outlined here have been constructed based on a comprehensive review of world literature. The following bibliographic databases were searched: MEDLINE, Scopus, and CINAHL. The search terms included but were not limited to “bladder pain syndrome,” “interstitial cystitis,” and “treatment.” International guidelines and the chronic care model (CCM) were also evaluated [9, 11]. The approach outlined is multidisciplinary and includes urologists, specialist nurses (urotherapists), and gynaecologists with an extended network to involve pain specialists, primary care physicians (PCPs), and psychologists.

### 2.1. The Challenge of BPS

The first step for clinicians involved in the care of BPS is an appreciation and understanding of why it is such a challenging condition to treat. From uncertainty regarding pathophysiological causation, disagreement in disease definition, heterogeneity in clinical phenotypes, to the lack of pathognomonic investigations and limitations in treatment efficacy, each part is contentious and renders BPS to hold an enigmatic status. Transparency and honesty with the patient about this from the outset are recommended to manage and set realistic expectations. Yeh et al. recorded outcomes of BPS patients at long-term follow-up (mean duration 16.6 ± 9.75 years) [12]. Only 12% achieved a status of being symptom-free, and 47% reported an improvement of at least 50% compared with the baseline. In a small number (6%), their condition continued to worsen despite treatment. Therefore, patients need to be counselled carefully and in a sensitive manner. The patients should be advised that they will be embarking on a journey, which can be both long and frustrating. More than one-third of patients receive at least four different treatment types. Such are the frustrations associated with having a “pain that cannot be seen,” and many patients with BPS disengage with medical care despite having ongoing symptoms [13]. The multifactorial nature of BPS demands a management pathway, which reflects this. A dedicated multidisciplinary team (MDT) meeting is recommended to discuss patients as required. The patients can be reassured that a range of healthcare professionals will be involved in their care [10]. A survey of PCPs' knowledge of BPS revealed that only 61% correctly answered that it was not caused by a psychiatric illness [14]. Consultations for BPS generally demand more time and so it is worth scheduling the clinic time to be longer than normal if possible.

### 2.2. Presentation

While BPS can affect males, up to 90% of sufferers are females [15]. Berry et al. determined that females diagnosed with BPS are more likely to be multiparous and unmarried [2]. Over 90% of patients with BPS are Caucasian, and the average age at diagnosis is the fourth decade of life [10]. The delay from symptom onset to diagnosis is common and is estimated to be 3 to 7 years [16]. Medical records of patients with BPS reveal a significantly higher number of visits to healthcare providers [4]. The International Continence Society (ICS) defines BPS as “persistent or recurrent chronic pelvic pain, pressure or discomfort perceived to be related to the urinary bladder accompanied by at least one other urinary symptom such as an urgent need to void or urinary frequency” [17]. Several other definitions exist; however, common to all of them are themes of chronicity, pelvic pain, and accompanying bothersome lower urinary tract symptoms (LUTS) [10]. Regarding the latter, urinary frequency, urgency, and nocturia are the most commonly reported. Ito et al. reported that the urinary frequency was reported in 98.3% of patients [18]. Typically, the urinary frequency associated with BPS ranges from 8 to 50 times a day. While urinary urgency is common, urinary incontinence (UI) is not. Urinary urgency associated with BPS is driven by the principal need to void in order to stop pain rather than to prevent leakage [8]. Pain is a hallmark symptom, and indeed, BPS is now classed as a subdivision of the chronic pelvic pain umbrella according to the European Association of Urology (EAU) guidelines [7]. The patients may report pain in all parts of the pelvic region, and it may be associated with triggers such as specific foods/beverages and/or sexual intercourse. The patients may have been previously managed with several courses of antibiotics in the community but to no avail. The constellation of burdensome symptoms associated with BPS results in an estimated 42% of patients not working and 18% of patients only working part time [19]. The studies investigating the natural history of BPS reveal that the symptom burden progressively worsens over a period of 3 to 5 years and reaches a plateau thereafter [20]. Late deterioration of symptoms is rare [10]. The psychological impact is pervasive, and patients are likely to have concomitant anxiety (14–52%) and/or depression (16–70%) [20].

### 2.3. Diagnosis

#### 2.3.1. History

Historically, diagnostic criteria such as those developed by the National Institute of Diabetes and Digestive and Kidney Diseases (NIDDK) included objective stigmata such as cystoscopic confirmation of diffuse glomerulations or Hunner's lesions (HLs) as well as certain cystometric properties such as a bladder capacity of less than 350 ml when awake [21]. A paradigm shift has taken place towards a diagnosis established on the presence of the abovementioned symptom constellation (Table 1). A detailed history is therefore required to elicit the symptomatology. Regarding family history, twin studies reveal that there is likely a genetic component to BPS, but this remains poorly understood [22].

#### 2.3.2. Examination

All patients should undergo a physical examination. This should include the abdomen and external genitalia as well as bimanual pelvic examination in women. Strength, tone, and point tenderness of pelvic floor muscles should be documented [6]. Urologists may be less familiar with the intricacies of this particular examination, and additional training from gynaecology colleagues can be valuable. Focused neurological examination in all patients to rule out occult disease should be performed, as well as a digital rectal examination (DRE) in men [23].

#### 2.3.3. Useful Adjuncts

Such is the difficulty associated with the bladder being “an unreliable witness,” and supplementation of the history through validated questionnaires is beneficial [24, 25]. The symptom areas are highlighted, which are burdening the patient the most, and they offer a means of grading severity. They also serve as a baseline assessment for comparison after treatment. There are many patient-reported outcome measures (PROMs) used in the setting of BPS including the O'Leary-Sant interstitial cystitis symptom index (ICSI), Bladder Pain/Interstitial Cystitis Symptom Score (BPIC-SS), and Pain, Urgency, Frequency (PUF) score. There are also several relevant modules from the International Consultation on Incontinence Questionnaires (ICIQs) such as Female Lower Urinary Tract Symptoms (ICIQ-FLUTS) and Lower Urinary Tract Symptoms Quality of Life Module (ICIQ-LUTSqol), which are available in several languages [26]. A bladder diary can also be issued to the patient to complete. It is worthwhile for centres to consider assembling a package of such assessment tools, which can be distributed to the patient in advance of the consultation [27].

### 2.4. Investigations

#### 2.4.1. Simple Tests

**Figure 1:**
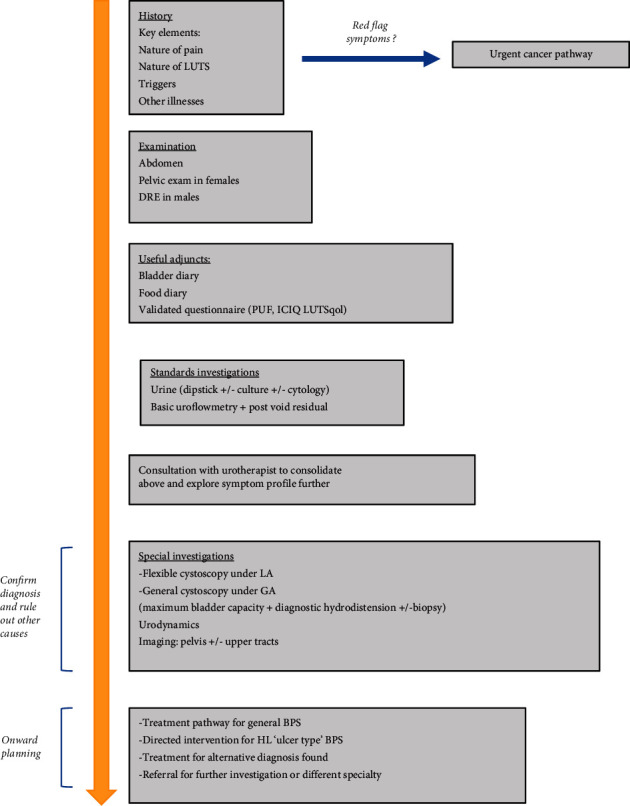
Diagnostic pathway for BPS.

On arrival to the clinic, patients should undergo urine dipstick (+/- culture) analysis and uroflowmetry including measurement of post-void residual (PVR) urine (Figure 1). Performing a urine culture can sometimes be considered in patients with negative dipstick to measure for a clinically significant bacteria burden, which may not reach standard thresholds for diagnosis [27, 28]. Communication with a local microbiologist is recommended to explore this service. Those with relevant risk factors such as a history of smoking should be considered for urine cytology testing. The patient's electronic records should be studied to reveal the results of any previous urine cultures and recent blood tests. If the history has revealed any red flag symptoms such as visible haematuria or abnormal prostate on DRE, patients should be diverted to the appropriate oncological pathway.

#### 2.4.2. Special Tests

While no single investigation can confirm a diagnosis, there are several additional tests, which can be considered. These can help support the diagnosis of BPS and rule out other conditions. Flexible cystoscopy under local anaesthetic (LA) can be performed to do the latter. However, rigid cystoscopy under general anaesthetic (GA) is needed to measure maximum bladder capacity and perform hydrodistension to observe for glomerulations. While glomerulations were previously considered an automatic criterion for BPS, this is no longer the case, as more recent research has revealed that glomerulations can also be present in healthy, asymptomatic individuals. For diagnosing BPS, a previous meta-analysis by Wennevik et al. revealed that glomerulations only carry a sensitivity and specificity of 60% and 62%, respectively [29]. Cystoscopy may also reveal HLs, which typically display a central pale scar with a stellate appearance and are surrounded by reddened mucosa [30]. Biopsy shows characteristic inflammation with lymphoplasmacytic cells and increased vascularity and epithelial denudation [31]. The presence of HLs is also no longer a prerequisite for BPS diagnosis, and findings from the IC Database study showed that only 11.7% of women had HLs [32]. However, this endoscopic finding does categorise the patient into a specific phenotype termed “Ulcer-type” BPS [31]. Urodynamic studies (UDS) can be considered to help distinguish from other disorders such as overactive bladder (OAB), especially where patients have shown no response to first-line treatments. Kim et al. determined patients with BPS to have an earlier first sensation of filling and a smaller cystometric capacity compared with OAB subjects, which is consistent with previous research [33]. UDS can also help investigate bladder outflow obstruction and poor detrusor contractility [34]. Of note, bladder filling during UDS in the context of BPS can be particularly uncomfortable for the patient. Pelvic and/or upper tract imaging can be considered by the clinician if it will help guide the diagnosis.

#### 2.4.3. Diagnostic Tests No Longer Part of Clinical Practice

In accordance with most guidelines, the authors do not perform the potassium sensitivity test as it can be extremely uncomfortable for the patient and even trigger symptom flares. In addition, the predictive value is low [7]. Intravesical anaesthetic challenge test with alkalinised lidocaine is no longer part of common clinical practice as it is supported by limited evidence [6]. Urinary biomarkers are a promising area of research in precision medicine but are not yet in clinical use [35].

#### 2.4.4. Differential Diagnosis

There are many alternative diagnoses to consider during the investigation (Table 1). As reflected by the changes in terminology for BPS, central to the diagnosis of BPS is excluding other causes. Both the history and many of the investigations help serve to eliminate these alternatives. In all patients, a priority is to exclude a urological malignancy. Benign urological conditions to consider include urinary tract infection (UTI) and stone disease. Gastrointestinal symptoms should prompt the clinician to consider conditions such as diverticular disease and inflammatory bowel disease (IBD). While less common, the possibility of a neurological cause, e.g., multiple sclerosis, should not be overlooked. Certain conditions are gender-specific. In females, this includes gynaecological pathologies such as endometriosis and pelvic inflammatory disease (PID). In males, chronic prostatitis and bladder outflow obstruction are differential diagnoses.

### 2.5. Treatment

#### 2.5.1. Initial

A pivotal approach to BPS is the early involvement of an urotherapist who can play a key diagnostic and therapeutic role [36]. In the Scandinavian setting, urotherapists are specialist nurses with a university-accredited qualification in bladder dysfunction. It allows the patient's history to be explored in a supportive environment but also one of great experience in this area. A conservative treatment approach is always adopted first, and the establishment of realistic expectations is entered into early with the patient. Lifestyle modifications should be recommended such as the promotion of regular exercise and dietary modifications including reduction in caffeine intake and other common aggressors such as spicy foods, alcohol, and citrus fruits. This should be combined with counselling regarding smoking cessation. The patient's existing pain management strategies need to be addressed, and it is not uncommon for BPS patients to have adopted self-initiated remedies such as nonprescribed medication(s). The use of illicit drugs should prompt the clinician to rule out a possible diagnosis of ketamine cystitis. The cases of particularly complex pain should be considered for early referral to a specialist pain team. Written guidance can be provided to the PCP in case of future symptom flares in the community. This may be particularly useful where patients live in remote geographic locations. Several of the instillation therapies are now able to be self-administered at home.

Involvement of the patient's partner/family can be helpful during the consultation. Research shows that this can help sufferers deal with the disease [37]. Such is the diverse and often misleading information that is freely available to patients reading online about BPS, and written information about BPS can be provided to aid patient education. BPS can be lonely for the patient to experience and so the patient can be informed of support groups. These groups also often provide information on alternative and complementary therapies such as acupuncture, reflexology, and hypnotherapy. Symptoms can be worsened by stress, and therefore, exploring behavioural modifications can be of benefit and consideration for a talking therapy such as cognitive behavioural therapy (CBT). This can be facilitated by the PCP. Where the physical examination has elicited pelvic floor tenderness or trigger points, manual physical therapy can be initiated and the involvement of physiotherapist with relevant expertise may be warranted.

#### 2.5.2. Oral Treatments

**Figure 2:**
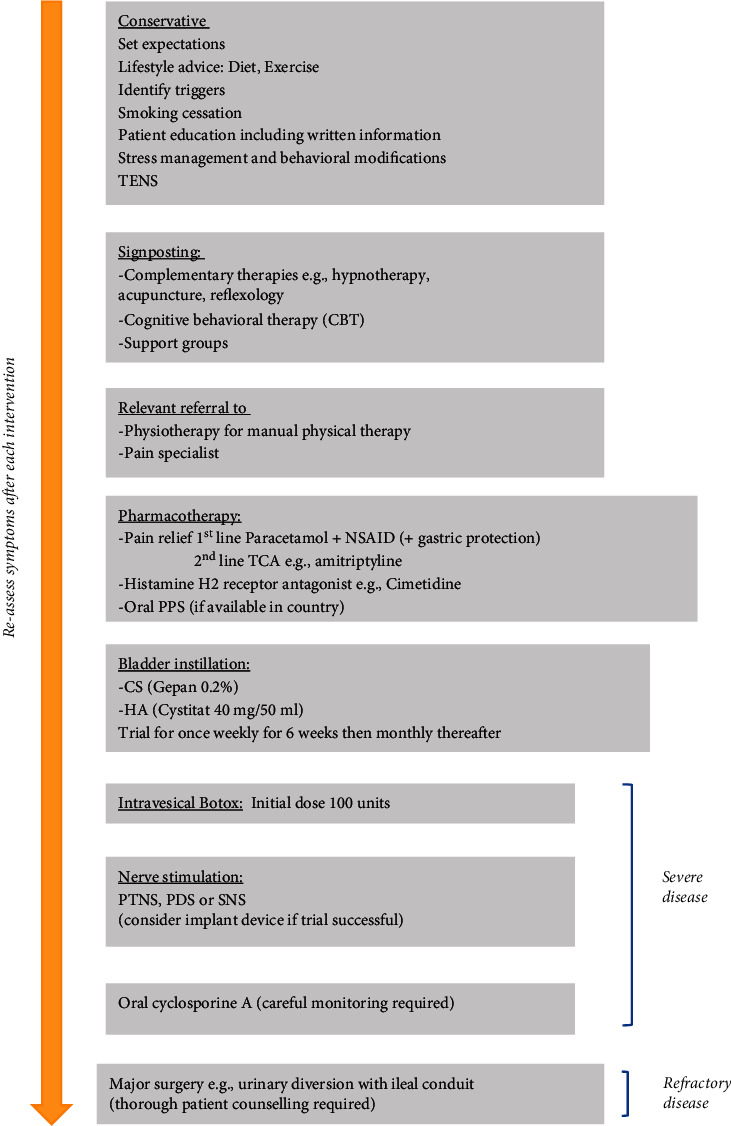
Treatment pathway for BPS.

Oral pharmacotherapy forms the mainstay of the next step where conservative approaches have failed (Figure 2). Nonsteroidal anti-inflammatory drugs (NSAIDs) are the standard analgesia to begin with (and regular gastric protection) together with paracetamol. Tricyclic antidepressants (TCAs) such as amitriptyline are another option for treating pain and helping to ameliorate the burden of storage LUTS. Cimetidine, a histamine H2 receptor antagonist, can also be offered. This takes effect owing to a similar peptidergic pathway in the bladder [38].

#### 2.5.3. Instillation Treatments

There are a number of instillation therapies available worldwide including chondroitin sulfate (CS), hyaluronic acid (HA), heparin, lidocaine, pentosan polysulfate sodium (PPS), and dimethyl sulfoxide (DMSO). The majority of these represent replenishment strategies to restore the architecture of the glycosaminoglycan (GAG) layer and the natural protection it provides [39]. Their clinical implementation is largely dependent on the licensing it receives in a particular country. In the European setting, chondroitin sulfate (CS) and hyaluronic acid (HA) are two of the most commonly used agents. A typical regime is once weekly for six weeks and monthly thereafter as required. HA and CS are also available as a combination (iAluRil ®). The commonest adverse events (AEs) are pain, irritation, and UTI. There is no strong evidence to recommend the superiority of any particular instillation therapy [39]. However, PPS and DMSO demonstrate worse side effect profiles (including headache and dizziness) and the need for ophthalmologic surveillance due to the risk of lens opacification [4].

#### 2.5.4. Intradetrusor Injection of Botulinum Toxin Type A (BoNT-A)

BoNT-A is an established treatment option for detrusor overactivity; however, it can also be considered in patients exhibiting a poor response to instillation treatments [41]. The standard initial dose is 100 units, which is consistent with most trials [41]. Cystoscopy (LA or GA) is required to deliver and inject the agent at approximately 10–20 sites. Patients are administered an initial dose, and maintenance can be given according to response. The patients must be counselled regarding the risk of urinary retention and need to learn self-catheterisation.

#### 2.5.5. Nerve Stimulation

Neuromodulators such as transcutaneous electrical nerve stimulation (TENS) work based on the gate control theory [42]. Simultaneous inputs are provided in large, myelinated nerve fibers, which serve to eliminate painful stimuli. TENS is a noninvasive and external device, which can be applied by the patient at home. This is a conservative treatment option. More invasive options are percutaneous tibial nerve stimulation (PTNS), pudendal nerve stimulation (PNS), and sacral nerve stimulation (SNS). These should only be considered in the later stages of treatment. These are available as permanent implants and are suitable options for patients with a good response to treatment with a temporary device. While success rates as high as 80% have been reported, only limited evidence exists to support the application of such implants for BPS [6]. However, careful counselling is required regarding AEs such as infection and the need for revision or removal. Both BoNT-A and invasive neuromodulators are categorised as fourth-line treatments in international guidelines; however, there is no recommendation beyond this to guide, which of these treatments should be tried first. However, BoNT-A is arguably the most natural next step to begin with after the other intravesical treatments have been trialed. On a practical level, it is also easier to offer this service compared with neuromodulation.

#### 2.5.6. Oral Cyclosporine A

Oral cyclosporine A, an immunosuppressive agent, is now available as a possible treatment option for patients with refractory disease [43]. Fastidious monitoring is required for AEs such as hypertension and renal failure. A clinically significant proportion of patients will discontinue treatment early due to these sequelae [6–9].

#### 2.5.7. Surgery

In those patients who have exhausted all other treatment options and whose symptoms significantly affect their quality of life, surgery can be considered. However, the decision for surgery requires careful patient counselling regarding morbidity (complications include infection, bowel obstruction, ureteric stricture, and stoma problems) and mortality associated with the surgery. It must also be explained that there is no guarantee that surgery will relieve their pain. Other long-term considerations for the patient include impact on sexual function, body image, and lifestyle [44]. The patient must understand that they are potentially swapping one set of problems for another. Options include urinary diversion with ileal conduit or a continent urinary diversion. The decision for whether a continent urinary diversion is opted for is influenced by factors such as patient preference and history of previous surgery and irradiation [45]. Urinary diversion can be done with or without cystectomy. However, there exists controversy surrounding whether it is permissible to leave the “diseased organ” in situ. As highlighted by the ICS, it appears that for many patients, the result of their native bladder no longer storing urine is sufficient to yield satisfactory symptom improvement. Pyocystis is reported to occur between 3.3 and 67% of cases [46].

#### 2.5.8. Interventions for HLs, “Ulcer-Type” BPS

Endoscopic identification of HLs should prompt a directed treatment strategy. The removal of the scarred urothelial tissue can be achieved through transurethral resection (TUR) and coagulation/fulguration. In a series of 59 patients, 13.1% and 57.2% required repeat intervention at 12 months and 24 months, respectively [47]. Triamcinolone acetonide (TA), a long-acting synthetic steroid, can be endoscopically injected into the lesion [48]. This allows direct administration of the steroid compared to treatment with oral steroids, which is systemically absorbed. The latter is not recommended due to AEs such as hypertension and diabetes mellitus. Evidence is largely limited to small case series such as that by Funaro et al. [49]. Measuring pain using a Likert scale (0–10), it improved significantly at one-month follow-up (8.3 vs. 3, *p* < 0.01) and the average time before re-treatment was 345 days.

#### 2.5.9. Treatments Not Recommended

Several treatments are no longer recommended in contemporary clinical practice. This includes intravesical Bacillus Calmette-Guerin (BCG), which is not used given that the side effects outweigh any potential therapeutic gains [9]. Long-term antibiotics are not advocated due to a lack of proven efficacy, side effects, and antimicrobial resistance (AMR). Long-duration (>10 mins) and high-pressure (>80 to 100 cm H_2_0) bladder distension has not been shown to improve outcomes and can be associated with serious complications such as bladder perforation [6].

## 3. The Role of the Urologist

Given the multidisciplinary approach, which is recommended in the management of BPS, the urologist is often in a unique position to act as the vehicle to coordinate such efforts and establish a local network of health professionals with relevant expertise. This is enhanced if individual departments can develop a treatment algorithm, which is tailored to the services they can offer. Having a nominated urologist to champion and lead such an initiative can complement this. Where local services for treatment services do not exist, e.g., urinary diversion or nerve stimulation, developing a referral pathway to a specialist centre is recommended. Maintaining communication with primary care teams is also valuable to update referral criteria, support for patient care, and provide educational events.

## 4. Limitations and Future Research

This review does not serve as an exhaustive guide to BPS management. However, it does offer pragmatic recommendations for this clinical challenge. Randomised studies are warranted to strengthen the evidence basis for many of the treatments and development of a universally agreed core outcome set [50].

## 5. Conclusion

BPS is a complex condition and is challenging for clinicians to manage. Diagnosis in the current era is established on the basis of clinical symptoms rather than pathognomonic criteria. Treatment follows a stepwise and multimodal approach. Extirpative surgery is offered but only in cases of refractory disease and must be supported by thorough preoperative counselling.

## Figures and Tables

**Table 1:** Characteristics of BPS.

Risk factors	Presenting symptoms	Diagnoses to exclude	Common coexisting conditions
Female gender Age Depression Smoking history High intake caffeine Lower socioeconomic Group Multiparity Lower socioeconomic group	*Core*: Pain Frequency Urgency N.B chronic duration. *Additional*: Nocturia Dyspareunia Anal discomfort	*Males and females*: Urinary tract infection Malignancy Urinary stone disease Overactive bladder Pelvic adhesions Inflammatory bowel disease Hernia Genital herpes Cystitis caused by radiation, tuberculosis, or chemicals, e.g., cyclophosphamide Ketamine bladder Pudendal neuropathy Multiple sclerosis Spinal cord injury Diverticular disease (of the bowel) *Females*: Endometriosis Pelvic inflammatory disease Vaginitis Eroded mesh Urethral diverticulum Urogenital prolapse Pregnancy Pelvic floor muscle tightness *Males*: Chronic prostatitis Bladder outflow obstruction	Fibromyalgia Irritable bowel syndrome Vulvodynia Sjogren's syndrome Chronic headache Chronic fatigue syndrome Depression Anxiety

## Data Availability

No data were used to support this study.
